# Hybrid SVM-CNN Classification Technique for Human–Vehicle Targets in an Automotive LFMCW Radar †

**DOI:** 10.3390/s20123504

**Published:** 2020-06-21

**Authors:** Qisong Wu, Teng Gao, Zhichao Lai, Dianze Li

**Affiliations:** Key Laboratory of Underwater Acoustic Signal Processing of Ministry of Education Southeast University, Nanjing 210096, China; 220170937@seu.edu.cn (T.G.); ghu@seu.edu.cn (Z.L.); 220190771@seu.edu.cn (D.L.)

**Keywords:** millimeter-wave radar, convolutional neural network, human–vehicle classification

## Abstract

Human–vehicle classification is an essential component to avoiding accidents in autonomous driving. The classification technique based on the automotive radar sensor has been paid more attention by related researchers, owing to its robustness to low-light conditions and severe weather. In the paper, we propose a hybrid support vector machine–convolutional neural network (SVM-CNN) approach to address the class-imbalance classification of vehicles and pedestrians with limited experimental radar data available. A two-stage scheme with the combination of feature-based SVM technique and deep learning-based CNN is employed. In the first stage, the modified SVM technique based on these distinct physical features is firstly used to recognize vehicles to effectively alleviate the imbalance ratio of vehicles to pedestrians in the data level. Then, the residual unclassified images will be used as inputs to the deep network for the subsequent classification, and we introduce a weighted false error function into deep network architectures to enhance the class-imbalance classification performance at the algorithm level. The proposed SVM-CNN approach takes full advantage of both the locations of underlying class in the entire Range-Doppler image and automatical local feature learning in the CNN with sliding filter bank to improve the classification performance. Experimental results demonstrate the superior performances of the proposed method with the $F_{1}$ score of 0.90 and area under the curve (AUC) of the receiver operating characteristic (ROC) of 0.99 over several state-of-the-art methods with limited experimental radar data available in a 77 GHz automotive radar.

## 1. Introduction

In recent years, autonomous vehicles have received widespread attention from the academic research community and the general public. It has become a critical research topic in autonomous driving to perceive the surrounding environment and other moving entities, such as vehicles, humans or animals, to protect vulnerable road users [1,2]. Autonomous vehicles obtain a semantic understanding of the scenes through different sensors in practical applications, which is vital to avoiding unnecessary evasive/emergency brake maneuvers for harmless objects [3]. Radar sensor is regarded as one of key components in autonomous driving, owing to robustness to low-light conditions and severe weather. The millimeter-wave (MMW) radar with linear frequency modulated continuous wave (LFMCW) has become a prevailing trend in autonomous driving, since it has the capability of distinguishing multiple targets due to high range resolution and extracting the object’s motion characteristics for classification [4]. However, it is still an open research area for the applicability and adaptation of these techniques in the specific automotive context. In particular, significant researches in the context of automotive radar have been devoted to the issue of accurately detecting and classifying pedestrians to preserve their safety.

A great number of efforts have been given to addressing the classification in the radar sensor. A two-stage target classification system with reported over 90% accuracy was proposed when distinguishing vehicles and pedestrians. It extracts features from micro-Doppler (MD) signatures, and the additional features calculated from the tracker are fed back to the target recognition system to improve the system performance [5]. Schubert looked at using Range-Doppler images as the domain to perform classification. They coped with the clustering of all these reflections into appropriate groups to extract features that are classified in a support vector machine (SVM) classifier [6]. Further works focused on using different domains of information to achieve vehicle and pedestrian classification, such as the phase characteristic of object signature [7], and the defined parameter, root radar cross section (RRCS) [8]. Those methods mentioned above required ‘handcrafted’ extraction process on the radar data in order to obtain the most suitable combination of features to maximize classification accuracy [9,10]. Nevertheless, the robustness of such features remains questionable, because the illumination of the wheels by radar strongly depends on the orientation of the vehicle to the sensor. Therefore, it is not well applied in achieving reliable automatic classification in the diversity of scenarios. Recently, an emerging stream of work in the literature has used neural networks as a processing tool to bypass the feature extraction step and adaptively select the most suitable features, and they have improved the state-of-the-art in speech recognition, computer vision, and other domains [11]. Further work on the use of convolutional neural networks (CNNs) in the context of human activity recognition for assisted living has been presented in the radar systems [12,13]. Kim introduced a drone classification method based on CNN and new image data for CNN, merged Doppler image, which concatenated micro-Doppler signature and the cadence–velocity diagram in one image to find the features in the Doppler signal from both time and frequency domains [14]. Hadhrami presented a classification approach of ground radar moving target, which utilized a pre-trained CNN as feature extractors whereas the output features were used to train a multiclass support vector machine (SVM) classifier [15]. Angelov presented results for the classification of different classes of targets using automotive radar data [16]. Kim proposed a method to simultaneously detect and classify objects by using a deep learning model, specifically you only look once (YOLO), with pre-processed automotive radar signals [17]. Other works have looked at classifying different human gaits in the context of area surveillance using ground-based radar, in particular identifying individual pedestrians as opposed to a group of multiple people, either using CNNs or recurrent neural networks (RNNs) on the spectrograms [18,19].

In a practical automotive context, a representative scenario is that a vehicle mounted by an automotive radar sensor runs on the highway. In this case, it often occurs that the vast majority class of detections are vehicles, that is to say, the number of vehicles among detections is much more than the number of pedestrians. It would lead to a typical class imbalance issue in the practical context. Researches’ main ways of addressing this problem are categorized into three classes including data-level methods, algorithm-level techniques and hybrid approaches [20]. In general, data-level methods modify the training distributions by sampling to decrease the level of imbalance [21,22,23]. Hensman provided that balancing the training data with random over-sampling (ROS) can improve the classification of imbalanced image data [21], and the ROS method randomly duplicates minority samples until all classes in the training set have an equal number of samples. Pouyanfar introduced a new dynamic sampling method that adjusts sampling rates according to class-wise performance [22]. Reference [23] showed the network less of what it has already learned and more of what it does not understand yet by over-sampling the low performing classes and under-sampling the high performing classes. Algorithm-level methods for handling class imbalance, commonly implemented with a weight or cost schema, include modifying the underlying learner or its output in order to reduce bias towards the majority group. Lin introduced new loss functions that allow the minority samples to contribute more to the loss, and reshaping the standard cross entropy loss was proposed to effectively capture classification errors from both majority class and minority class equally [24]. Wang employed a cost-sensitive deep neural network (CSDNN) method to detect hospital readmission, and they concatenate the features extracted by the convolutional neural network (CNN) and categorical embeddings, forming a final input feature vector that is fed to a deep neural network (DNN) for classification [25]. Hybrid systems strategically combine both sampling and algorithmic methods. One strategy includes performing data sampling to reduce class noise and imbalance, and then applying cost-sensitive learning or thresholding to further reduce the bias towards the majority group [26,27].

In this paper, a hybrid SVM-CNN approach is proposed to address the class-imbalance classification of vehicles and pedestrians with limited experimental radar data available in an automotive radar sensor. A two-stage scheme with the combination of feature-based SVM technique and deep learning-based CNN is employed. In contrast to optical images, the Range-Doppler images based on the LFMCW radar sensor have the capability of revealing the information of target’s range and velocityp, which are significantly useful physical features for the classification of pedestrians and vehicles. In the first stage, a feature-based modified SVM technique is used to recognize vehicles with the precision of 1. The role of this first stage lies in two aspects. On the one hand, the distinct physical feature like high velocity, which corresponds to positions in the Range-Doppler image, may lead to a simple classification for vehicles. However, the convolution operation in the CNN is used to slide the filter bank with a small size across the input, producing activations at each receptive field that combine to form a local feature map, and this operation is often invariant to location. On the other hand, a number of vehicles are recognized in the first stage by using the feature based SVM technique, and it would effectively alleviate the imbalance ratio of vehicles to pedestrians at the data level. The residual unclassified images will be used as inputs to the CNN for the subsequent classification in the second stage. To address the classification in the class-imbalance case, we introduce a weighted false error (WFE) function that can be deployed readily in deep network architectures, and take full advantage of the powerful ability of automatic feature learning in the CNN to improve the classification performance in the algorithm level. Experimental results demonstrate the superior performances of the proposed method over several state-of-the-art methods with limited experimental radar data available in a 77 GHz automotive radar sensor.

The remainder of this paper is organized as follows. Section 2 introduce the signal model in the LFMCW radar sensor. Section 3 presents the proposed hybrid SVM-CNN classification method in detail. Experimental results are shown in Section 4 to verify the effectiveness of the proposed method. Finally, Section 5 concludes this paper.

## 2. Signal Model in the LFMCW Radar Sensor

In an LFMCW radar sensor, the transmitted signal $s_{r}\left(t\right)$, which is referred to as a chirp waveform [28], is expressed as

(1) $$s_{r}\left(t\right)=A_{T}\exp\left(j2\pi \left(f_{c}t+\frac{\gamma t^{2}}{2}\right)\right),$$

where $A_{T}$ is the amplitude of the transmitted signal, and $f_{c}$ is the carrier frequency. γ=B/T is the chirp rate with the transmitted bandwidth of *B* and the sweep time of *T*, and t∈(0,T) is the fast-time.

According to Equation (1), the received signal s(t,τ) can be written by

(2) $$\begin{array}{cc} s(t,\tau ) & =\sum_{m=1}^{M}A_{m}\exp\left(j2\pi f_{c}\left(t-\frac{2R_{m}\left(\tau \right)}{c}\right)\right)\\ \; & \times \exp\left(j\pi \gamma {\left(t-\frac{2R_{m}\left(\tau \right)}{c}\right)}^{2}\right), \end{array}$$

where τ is the so-called slow time, $A_{m}$ is the amplitude of the *m*-th scatterer. $R_{m}\left(\tau \right)$ is the distance from the sensor to the *m*-th scatterer, and *c* is the speed of electromagnetic wave propagation. *M* is the total number of scatterers.

The beat signal can be obtained by mixing the echo signal with the transmitted signal, and it can be expressed as,

(3) $$\begin{array}{cc} \chi (t,\tau ) & =s_{r}\left(t\right)s^{*}\left(t,\tau \right)\\ \; & \approx \sum_{m=1}^{M}A_{T}A_{m}\exp\left(j2\pi \gamma \frac{2R_{m}\left(\tau \right)}{c}t+j\frac{4\pi R_{m}\left(\tau \right)}{\lambda }\right), \end{array}$$

where $s^{*}\left(t\right)$ represents the conjugate of the received signal s(t), and $\lambda =c/f_{c}$ denotes the wavelength of the carrier frequency. The residual video phase is found to be negligible and can then be ignored due to relatively small time delays [29].

A close look must be given at the exponential terms in Equation (3). The first exponential term exhibits a constant frequency along the fast time, which is proportional to the target range. $f_{bm}\left(\tau \right)=2\gamma R_{m}\left(\tau \right)/c$ is just the beat frequency of the *m*-th scatterer. The second exponential term shows the phase of target with the range of $R_{m}\left(\tau \right)$, and its phase history $\phi_{m}\left(\tau \right)=4\pi R_{m}\left(\tau \right)/\lambda$ is related to the range evolution of $R_{m}\left(\tau \right)$ along slow time τ. For a stationary target, the range history $R_{m}\left(\tau \right)$ is a constant across chirps, and thus it has the zero Doppler. However, for the moving targets, like pedestrians or vehicles, the range history $R_{m}\left(\tau \right)$ would change across chirps and show the Doppler frequency deviated from zero, which is proportional to the velocity of moving targets. This is the main reason why the LFMCW radar has the capability of detecting moving targets and estimating their velocities.

Considering an LFMCW radar sensor with the sample rate of $f_{s}$ and the sweep time of *T*, we thus have the sampling number of $N=f_{s}T$ in each chirp. To acquire the range evolution of targets along the slow time, *L* chirps are used to form a “frame" in the system. The two-dimension discrete Fourier Transform (2D-DFT) are performed over Equation (3) to acquire a Range-Doppler image $Y\in C^{N\times L}$ as

(4) $$\begin{array}{c} \begin{array}{cc} Y(k,p)= & \sum_{l=0}^{L-1}\left[\sum_{n=0}^{N-1}\chi \left(n,l\right)w_{r}\left(n\right)\exp\left(-j2\pi \frac{n\cdot k}{N}\right)\right]\times w_{c}\left(l\right)\exp\left(-j2\pi \frac{l\cdot p}{L}\right), \end{array} \end{array}$$

where $w_{r}$ and $w_{c}$ are the window functions in the discrete fourier transform (DFT) procedure, respectively, k∈{1,⋯,N} and p∈{1,⋯,L}. The Gaussian function windows are often used both in range and cross-range dimensions. The Range-Doppler image will be used to perform the classification.

## 3. Proposed Hybrid SVM-CNN Classification Method

In this section, a novel two-stage hybrid SVM-CNN method will be introduced to acquire enhanced classification performance with mitigating the class-imbalance issue. Unlike these methods, which directly utilize Range-Doppler images or micro-Doppler images as input images into the neural network for the classification [12,13,16,18,19], we proposed a two-stage procedure with joint exploitation of the SVM and the CNN techniques. Before the SVM procedure, Range-Doppler image based data preprocessing is requisite for the subsequent classification.

### 3.1. Data Preprocessing

The aim of the data preprocessing is to find the potential targets under test. Data preprocessing includes the constant false alarm rate (CFAR) detection [30] and clustering of detections [31]. It is well-known that the CFAR method suggests whether the scattering points exist under the condition that the false alarm probability is kept constant, and cell averaging constant false alarm rate (CA-CFAR) is used for detection threshold estimation [30]. The positions of the potential target within Range-Doppler image, which represent the information of the range and the velocity, can be estimated and acquired. It is very important that physical features be used for the classification, and will be used in the feature based SVM procedure. In contrast to other sensor technologies suitable for pedestrian detection, like video, laser or ultrasonic, a significant advantage of radar is the ability to measure not only the position, but also the velocity of each scattering point.

Owing to high resolution in the LFMCW radar sensor, it is possible to detect a plenitude of scattering points reflected by one physical object. In subsequent steps in the data preprocessing, the cluster technique is then performed to map all these reflections to appropriate groups in order to exploit the advantages of multidimensional object size estimation or to extract features for classification purposes. The density-based spatial clustering of applications with noise (DB-SCAN) algorithm is often chosen for implementation due to robustness to noise or outlier. The principle of DB-SCAN is to identify groups of points by regions of different density based on the basic principle that the density inside of a cluster is higher than outside. After grouping relevant scattering points to appropriate clusters, their individual consistencies can be used to gather additional information about the nature of the underlying physical object. Measured target properties like spatial extension in range, spatial extension in velocity within Range-Doppler images are possible candidates to span a multi-dimensional feature space.

It is found that pedestrians and wheeled road traffic participants like vehicles show significant differences signatures in their range and velocity spreads within Range-Doppler images [32]. Rigid bodies like vehicles easily show a range spread signature due to relatively large sizes, whereas the spatial extension in velocity is quite narrow due to the identical velocity of body. However, the kinematical spread of a walking pedestrian changes periodically according to the gait cycle from nearly zero to approximately three times the mean walking velocity, and thus they often show a wider spread signature in velocity or Doppler than that in range. In addition, simple algorithms like comparison of the maximum velocity of a cluster with its kinematical centroid, respectively, its mean velocity may lead to an easy feature for object classification. It is the main reason why the feature-based SVM technique takes full advantage of these physical features for the vehicle classification, and is used in the first stage. In the data preprocessing, the selected feathers are provided in Table 1 according to [5]. However, the robustness of such a feature is questionable, because the illumination of the wheels by radar strongly depends on the orientation of the vehicle to the sensor. Therefore, a modified CNN is subsequently introduced for the enhanced classification in the class-imbalance case by exploiting the powerful capability of automatic selection of the most suitable features within the network itself. In the following subsection, the hybrid SVM-CNN classification method will be introduced.

### 3.2. Hybrid SVM-CNN Classification Method

#### 3.2.1. Modified SVM Approach

Given the selected feature vector and the corresponding class label, we have,

(5) $$\Psi =\left\{\left(x_{1},y_{1}\right),\left(x_{2},y_{2}\right),\cdots ,\left(x_{N},y_{N}\right)\right\},$$

where $x_{i}\in R^{5}$ is the feature vector of the *i*-th sample, $y_{i}\in \left\{-1,+1\right\}$ represents the class label of the *i*-th sample, and *N* is the size of training set Ψ. For the linearly separable dataset in a typical SVM method, there is only one hyperplane w·x+b=0 to separate positive and negative samples, with the maximum category interval. w is the feature weight and *b* is the bias on the hyperplane. The sample points on the hyperplane $\left|w\cdot x_{i}+b\right|=1$ are the support vectors, and the optimal solutions to the weight vector of w and the intercept of *b* are determined by these support vectors. The constrained optimization problem formed by the maximum category interval can be expressed as,

(6) $$\begin{array}{c} \begin{array}{cc} \min_{w,b}\; & \frac{1}{2}{∥w∥}^{2}\\ s.t.\; & y_{i}\left(w\cdot x_{i}+b\right)-1\geq 0.\;\;\;i=1,2,\cdots ,N \end{array} \end{array}$$

Furthermore, a slack variable $\xi_{i}\geq 0$ is introduced with the consideration of possible outlier labels in the dataset, and then the constraint becomes,

(7) $$y_{i}\left(w\cdot x_{i}+b\right)\geq 1-\xi_{i},$$

where $\xi_{i}=\max\left(0,1-y_{i}\left(w\cdot x_{i}+b\right)\right)$. Meanwhile, the slack variable loss is introduced into the objective function as,

(8) $$L\left(w,b\right)=\frac{1}{2}{∥w∥}^{2}+\eta \sum_{i=1}^{N}\xi_{i},$$

where η>0 is a penalty factor. The constrained optimization problem can be further expressed as,

(9) $$\begin{array}{c} \begin{array}{cc} \left\{\hat{w},\hat{b}\right\}=\arg\min_{w,b}\; & \frac{1}{2}{∥w∥}^{2}+\eta \sum_{i=1}^{N}\xi_{i}\\ s.t.\; & y_{i}\left(w\cdot x_{i}+b\right)\geq 1-\xi_{i}\;\;\;\xi_{i}\geq 0,\;i=1,2,\cdots ,N. \end{array} \end{array}$$

When the dataset is linearly inseparable, a kernel function can be used to transform the linearly inseparable problem into a linearly separable problem in a high-dimensional feature space. Then the Sequence Minimum Optimization (SMO) algorithm [33] can be used to address the convex quadratic programming problem in the SVM approach, and the optimal separating hyperplane with estimated parameters of $\hat{w}$ and $\hat{b}$ can be obtained.

In fact, it is difficult for only the standard SVM approach above to acquire satisfactory classification performance, since the illumination of the wheels by radar strongly depends on the orientation of the vehicle to the sensor, and these features of pedestrians and vehicles often overlap. In this paper, a further step for the learning of intercept of *b* will be performed to recognize vehicles by exploiting underlying prior information related with velocity and range. It is reasonable that the class with the velocity over a threshold of $v_{th}$ and the range extension over a threshold of $\rho_{th}$ is recognized as vehicles. This underlying prior information will be imposed to relearn the intercept of *b* to improve the precision of vehicles classification and alleviate the class-imbalance ratio. To address the relearning of *b*, we introduce the precision β, which is defined as [34],

(10) $$\kappa =\frac{{\mathrm{TP}}_{v}}{{\mathrm{TP}}_{v}+{\mathrm{FP}}_{v}},$$

where ${\mathrm{TP}}_{v}$ and ${\mathrm{FP}}_{v}$ are the abbreviations of true positive and false positive, respectively, and ${\mathrm{FP}}_{v}$ is the number of negative samples classified wrongly. It should be noted that the vehicle class in the SVM procedure is positive. According to (10), when the precision κ is equal to 1, it suggests that the vehicle samples are correctly classified, and no pedestrian (negative) sample is wrongly classified as positive (vehicle). The relearning of *b* aims at correctly recognizing vehicle class as much as possible with the precision κ=1 to alleviate the class-imbalance ratio. According to the prior information related with the velocity and range extensions, we relabel these samples that satisfy the requirements of thresholds of $v_{th}$ and $\rho_{th}$ as vehicle, and the class of the residual samples is pedestrian. We have relabeled sample data $\Psi^{\left(r\right)}$ as,

(11) $$\Psi^{\left(r\right)}=\left\{\left(x_{1},y_{1}^{\left(r\right)}\right),\left(x_{2},y_{2}^{\left(r\right)}\right),\cdots ,\left(x_{N},y_{N}^{\left(r\right)}\right)\right\},$$

where $y_{n}^{\left(r\right)}$ denotes the relabeled class of the *n*-th sample according to the prior thresholds. We relearn the intercept *b* with the given acquired weight vector $\hat{w}$ by the following optimization problem with the consideration of possible outlier labels,

(12) $$\begin{array}{cc} b^{*} & =\arg\min_{b}\;\sum_{i=1}^{N}\xi_{i} \end{array}$$

(13) $$\begin{array}{cc} s.t.\; & y_{i}^{\left(r\right)}\left(\hat{w}\cdot x_{i}+b\right)\geq 1-\xi_{i}\;\;\;\xi_{i}\geq 0,\;i=1,2,\cdots ,N. \end{array}$$

According to the steps above, the optimal weight vector $\hat{w}$ and the updated intercept of $b^{*}$ in the SVM approach are acquired to recognize vehicles as much as possible with the precision of 1. It is the capability of enhancing the accuracy of vehicle classification, and effectively alleviating the class-imbalance ratio in the next stage.

#### 3.2.2. Modified CNN Method

Recently, CNN has been successfully used in fields such as images, videos, text and writing text with radar sensors, and has shown powerful capability in classification and recognition [35,36,37,38,39,40,41]. The CNN technique with the mean square error (MSE) as the loss function has been verified as having significantly superior performance in the classification tasks. However, for data-imbalanced classification tasks, network weight updates being driven primarily by the majority class often tend to cause the majority class error to reduce rapidly at the expense of increasing minority class error [42]. It would lead to the classification performance deterioration due to a class-imbalance issue. Although the class-imbalance ratio of vehicles to pedestrians can be alleviated by the modified SVM approach in the first stage, this imbalance issue still exists and must be addressed in the second stage due to the limited recognized number of vehicles in the first stage.

**Figure 1:**
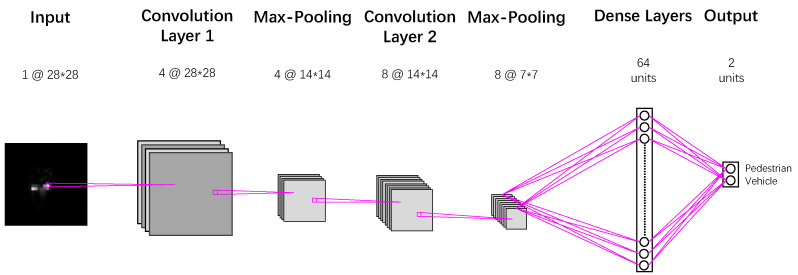
Convolutional neural network (CNN) architecture for the Range-Doppler image input. This CNN architecture with two convolutional and one fully-connected layer is kept identical for all experiments.

In the second stage, a downscaled CNN is used to classify the pedestrian and vehicle in the automotive radar sensor. Considering the relatively simple structures in the Range-Doppler images compared to the complex structures of objects in the natural images or videos, we downscale the standard VGG network with less layer and parameters. The employed network structure is shown in Figure 1. The whole network consists of two convolutional layers, two pooling layers, and one fully connected layer. Each convolutional layer is followed by a maximum pooling layer. Meanwhile, the dropout is employed in the fully connected layer to further avoid the data overfitting. Each convolutional layer applies the ReLU activation function, and the output layer adopts the Softmax function to obtain the confidence of each class, which is converted to the class label through the decision threshold.

In the process of networking training, the loss function is minimized to obtain the optimal network parameters by constantly iterating the training samples. The loss function can be generally defined as

(14) $$E\left(\theta \right)=L\;\left(c^{\left(i\right)},z^{\left(i\right)}\right),$$

where θ represents the network parameters vector, $z^{\left(i\right)}$ represents the one-shot encoding related with the label $y_{i}$ of the *i*-th sample. $z^{\left(i\right)}={\left[1,0\right]}^{T}$ is the pedestrian with $y_{i}=1$, $z^{\left(i\right)}={\left[0,1\right]}^{T}$ is the vehicle with $y_{i}=-1$. $c^{\left(i\right)}$ means the desired output probability vector of the *i*-th sample with the constraint $\sum_{k=1}^{n}c_{k}^{\left(i\right)}=1$. $c_{k}^{\left(i\right)}$ is the probability of the *k*-th category corresponding to the *i*-th sample, and n=2 is the number of categories in the binary classification. L(·) represents the loss function. The optimization objective function can be generally expressed as

(15) $$\theta^{*}=\arg\min_{\theta }\;E\left(\theta \right).$$

The MSE, which is defined as the mean square error between the class encoding z and the desired output c, is often employed as,

(16) $$\mathrm{MSE}=\frac{1}{N}\sum_{i=1}^{N}\sum_{k=1}^{n}\frac{1}{2}{\left(c_{k}^{\left(i\right)}-z_{k}^{\left(i\right)}\right)}^{2},$$

where *N* is the sample number of the training set. In the class-imbalance classification tasks, the loss function of MSE would not seem an optimal choice.

In order to highlight the importance of minority class, we introduce a modified loss function that places more attention on the impact of the minority class on network parameters besides MSE. To address this issue, we introduce the precision α and recall β as an additional evaluation metric, and express as [34],

(17) $$\alpha =\frac{{\mathrm{TP}}_{p}}{{\mathrm{TP}}_{p}+{\mathrm{FP}}_{p}},\;\;\;\;\beta =\frac{{\mathrm{TP}}_{p}}{{\mathrm{TP}}_{p}+{\mathrm{FN}}_{p}},$$

where ${\mathrm{TP}}_{p}$ represents the number of pedestrian samples classified correctly, ${\mathrm{FP}}_{p}$ indicates the number of vehicle samples wrongly classified as pedestrian, and ${\mathrm{FN}}_{p}$ denotes the number of misclassifications in the vehicle sample. It should be pointed out that pedestrian class is regarded as the positive one in the CNN procedure.

Since the precision and recall indexes pare trade-off, $F_{1}$ score is used to make a compromise between these, and can be defined as [34]

(18) $$F_{1}=2\cdot \frac{\alpha \cdot \beta }{\alpha +\beta },$$

where $F_{1}$ score is also defined as the harmonic mean of precision and recall. We introduce false precision error (FPE) to capture the precision error of samples classified positive, and express

(19) $$\begin{array}{c} \begin{array}{cc} \mathrm{FPE} & =\frac{1}{P}\sum_{i=1}^{P}\sum_{k=1}^{n}\frac{1}{2}{\left(c_{k}^{\left(p_{i}\right)}-z_{k}^{\left(p_{i}\right)}\right)}^{2}\\ s.t. & \;\begin{array}{cc} c_{0}^{\left(p_{i}\right)}\;> & \;c_{1}^{\left(p_{i}\right)}\\ z^{\left(p_{i}\right)}\;= & \;{\left[0,1\right]}^{T}, \end{array} \end{array} \end{array}$$

where $p_{i}$ indicates the sample indexes in which the samples with the negative label are predicted as positive. *P* is the number of errors in the samples classified as positive. we also employ false recall error (FRE), which denotes the recall error of positive samples, as

(20) $$\begin{array}{c} \begin{array}{cc} \mathrm{FRE} & =\frac{1}{R}\sum_{i=1}^{R}\sum_{k=1}^{n}\frac{1}{2}{\left(c_{k}^{\left(r_{i}\right)}-z_{k}^{\left(r_{i}\right)}\right)}^{2}\\ s.t. & \;\begin{array}{cc} c_{0}^{\left(r_{i}\right)}\;< & \;c_{1}^{\left(r_{i}\right)}\\ z^{\left(r_{i}\right)}\;= & \;{\left[1,0\right]}^{T}, \end{array} \end{array} \end{array}$$

where $r_{i}$ represents the indexes of the samples which are incorrectly classified in the positive samples. *R* means the number of misclassifications in the positive samples. The FE score is adopted to compromise FPE and FRE, and can be defined as,

(21) $$\mathrm{FE}=\frac{1}{F_{1}}=\frac{\frac{1}{\mathrm{FPE}}+\frac{1}{\mathrm{FRE}}}{2\cdot \frac{1}{\mathrm{FPE}}\cdot \frac{1}{\mathrm{FRE}}}=\frac{1}{2}\left(\mathrm{FPE}+\mathrm{FRE}\right).$$

The FE score can also be regarded as the average of FPE and FRE. By exploiting the FE score to balance the effect of minority class on the network training in the class-imbalance case, we modify the loss function L with the WFE (Weighted False Error) as

(22) $$E=w_{1}\cdot \mathrm{MSE}+w_{2}\cdot \mathrm{FE},$$

where $w_{1}$ and $w_{2}$ represent the weights of MSE and FE, respectively, and $w_{1}+w_{2}=1$ with $1\;\geq \;w_{1}$, $w_{2}\geq 0$, they make a compromise of MSE and FE. When $w_{1}=1$, the loss function reduces to the standard MSE. However, when $w_{2}=1$, the loss function is completely determined by the FE. By introducing the FE in the loss function, the effect of the minority class will be paid more attention to, compared to the loss function of only MSE.

### 3.3. Summary of Proposed Hybrid SVM-CNN Classification Method

**Figure 2:**
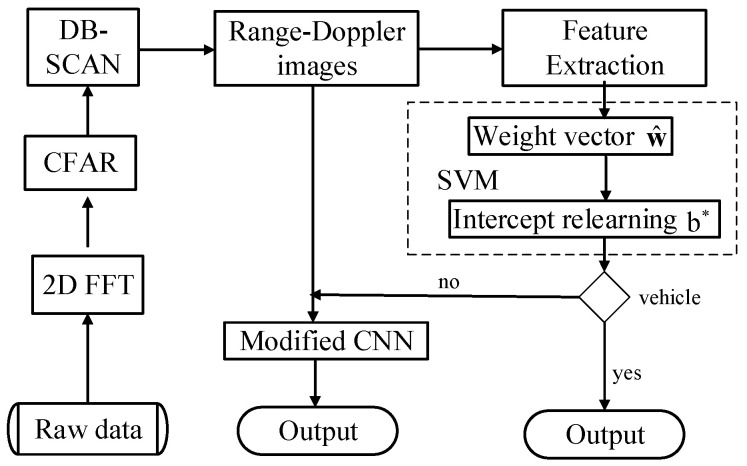
Flowchart of the hybrid support vector machine (SVM)-CNN classification method.

The flowchart of the hybrid SVM-CNN classification method is provided, as shown in Figure 2. In summary, the 2D-FFT operator is firstly performed over the raw data, and data preprocessing including CFAR and DB-SCAN is then used in the Range-Doppler images so that these physical features of underlying targets of interest are acquired for the subsequent classification. In the classification task, a two-stage scheme with the combination of featured based SVM technique and deep learning based CNN is employed. The modified SVM approach based on these distinct features, which involves the estimate of weight vector $\hat{w}$ and the relearning of the intercept $b^{*}$, is proposed to recognize vehicles to effectively alleviate the imbalance ratio of vehicles to pedestrian in the data level. The residual unclassified region-of-interest Range-Doppler images are used as inputs to the CNN. Then, the modified CNN approach is employed to acquire the enhanced classification results by introducing WFE into loss function in the class-imbalance classification task in the algorithm level.

### 3.4. Analysis of Computational Complexity

According to the flowchat of the hybrid SVM-CNN classification approach, the computational complexity involves the SVM and CNN procedures. The computational complexity of the standard SVM is $O(N_{s}^{2}+N_{s}DN)$ [43], where $N_{s}\ll N$ is the number of support vectors, *D* is the dimension of the input feature vector, and *N* is the total sample number. In the modified SVM approach, the estimate of weight vector $\hat{w}$ and the relearning of the intercept $b^{*}$ would take the computation of $O(2N_{s}^{2}+2N_{s}DN)$. With respect to the CNN in the second stage, it is known that the CNN is composed of convolutional layers, activation functions, pooling layers, and fully connected layers, and the main computational complexity lies in the multiplication operators in convolutional layers and fully connected layers. The overall computational complexity of the CNN approach is about $O(N\sum_{l=1}^{d}M_{l}^{2}K_{l}^{2}C_{l-1}C_{l})$ [44], where *d* is the network depth, $M_{l}$ represents the side length of the feature map in the *l*th convolutional layer, $K_{l}$ denotes the side length of the convolution kernel in the *l*-th convolutional layer, $C_{l}$ is the number of convolutional kernel in the *l*th convolutional layer, and $C_{l-1}$ is the number of convolutional kernel of the (l−1)-th convolutional layer. It is obvious that the computational complexity is much larger than that in the SVM method, the computational complexity of the proposed SVM-CNN mainly lies in that in the CNN stage. Therefore, the computational complexity of the proposed method is comparable to the standard CNN method.

## 4. Experiments

In this section, experimental results demonstrate the superior performance of the proposed hybrid SVM-CNN approach over several state-of-the-art methods. To quantify the classification performance, we introduce accuracy, precision, recall, $F_{1}$ score, and receiver operating characteristic (ROC) as performance indexes. All experiments were conducted on a 2.80 GHz PC using Python 3.0.

### 4.1. Datasets and Data Augmentation

**Figure 3:**
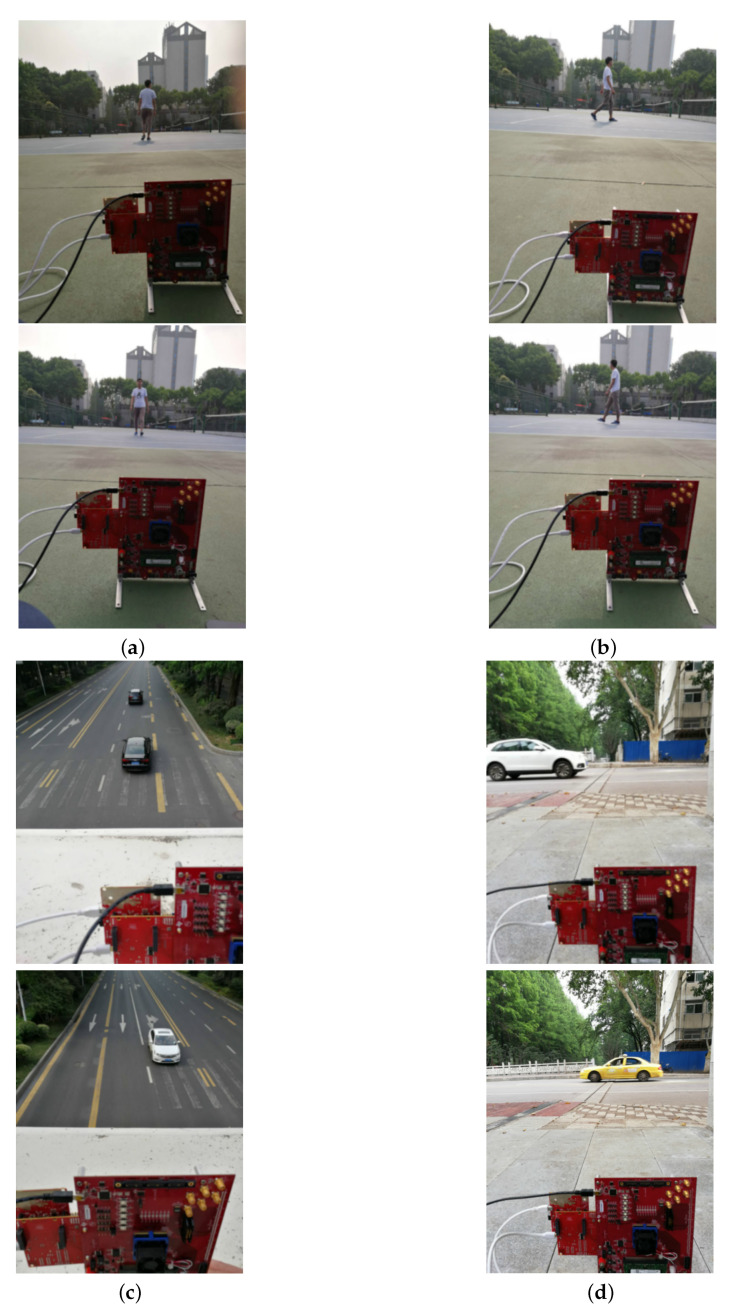
Typical motion scenes of pedestrian and vehicle. (**a**) pedestrian longitudinal movement (forwards and towards); (**b**) pedestrian lateral movement (left to right and right to left); (**c**) vehicle longitudinal movement (forwards and towards); (**d**) vehicle lateral movement (left to right and right to left).

In this experiment, a 77 GHz automotive radar sensor with the Texas Instruments (TI) IRW1443 evaluation board is used to perform experimental data collection [45], and radar parameters are shown in Table 2. In the experiments, the radar is fixed on the ground and received echo signals of moving targets in different motion scenarios for convenience. Typical examples of observed motion scenes are shown in Figure 3, and 600 data samples are collected with 500 vehicles and 100 pedestrians. Considering the relative small size of these effective samples, we enrich and extend the sample dataset by exploiting image preprocessing techniques including adjusting contrast, sharpness, saturation and SNR, and acquire the total 3000 samples with the invariant ratio of vehicles to pedestrians.

**Figure 4:**
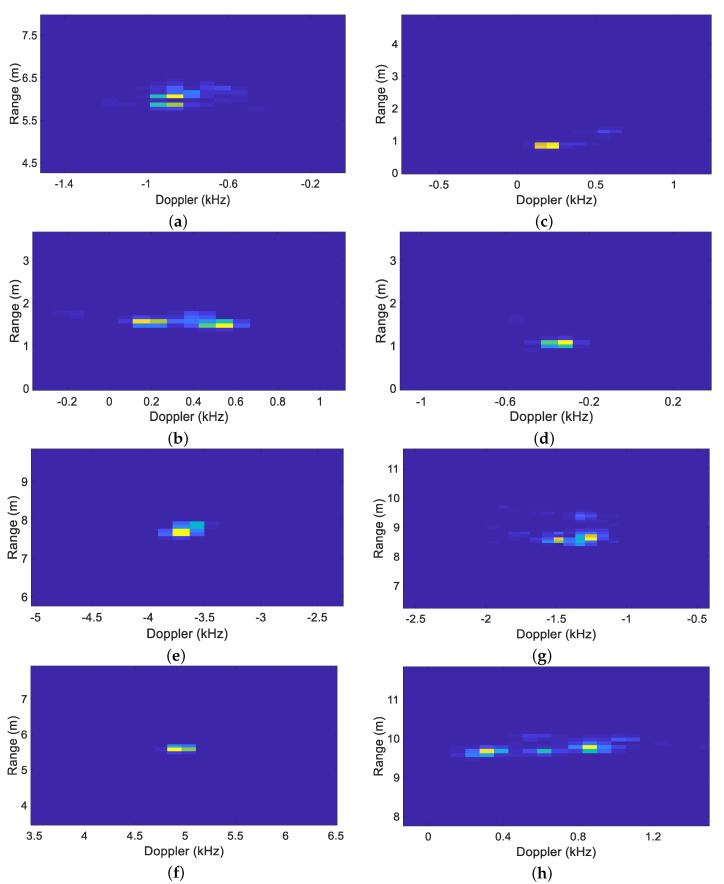
Range-Doppler images of pedestrian and vehicle. (**a**) pedestrian longitudinal movement (forwards); (**b**) pedestrian longitudinal movement (towards); (**c**) pedestrian lateral movement (left to right); (**d**) pedestrian lateral movement (right to left); (**e**) vehicle longitudinal movement (forwards); (**f**) vehicle longitudinal movement (towards); (**g**) vehicle lateral movement (left to right); (**h**) vehicle lateral movement (right to left).

We performed data preprocessing of echo data, and then obtain the Range-Doppler images and features for the SVM classifier. The Range-Doppler images of pedestrians and vehicles in diverse scenes are shown in Figure 4, and the extracted features are given in Table 1.

Since dimensional quantity of extracted features are quite different, we normalize the extracted features to improve the speed of convergence of the SVM classifier. The normalization formula is defined as

(23) $$\tilde{x}=\frac{x-\min(x)}{\max(x)-\min(x)}$$

where x represents the feature vector, $\tilde{x}$ is the normalized feature vector. ${\tilde{x}}^{\left(i\right)}$ means the *i*-th feature in the feature vector, and satisfies ${\tilde{x}}^{\left(i\right)}\in \left[0,1\right]$.

### 4.2. Classification Performance Comparisons

In the training process of the SVM classifier, 80% of the dataset is used for training and the remaining 20% is used for testing. In the experiments, the velocity threshold $v_{th}$ be 10 m/s and the range extension threshold $\rho_{th}$ is 2 m with the precision of 100%, and the improved $\hat{w},b^{*}$ can be obtained according to the modified SVM method. The relearned intercept $b^{*}$ is −4.08 compared to the standard intercept $\hat{b}$ of −0.81. The classification results of the SVM classifiers in the first stage are shown in Table 3. It is observed that the 483 vehicle samples are correctly recognized based on the modified SVM with the precision of 1, compared to the precision of 0.86 in the standard SVM in the training set. Although the number of correctly classified vehicle samples is smaller than that in the standard SVM in this stage, the modified SVM is capable to correctly recognize vehicles without pedestrian samples classified wrongly. In this stage, these vehicles, which have distinct physical features with high velocity and range extension, are easily recognized to improve the classification performance. On the other hand, it effectively alleviates the class-imbalance ratio of vehicles to pedestrian. The residual unclassified Range-Doppler images with the number of 1917 in the training set will put the neural network for the subsequent classification in the second stage.

**Figure 5:**
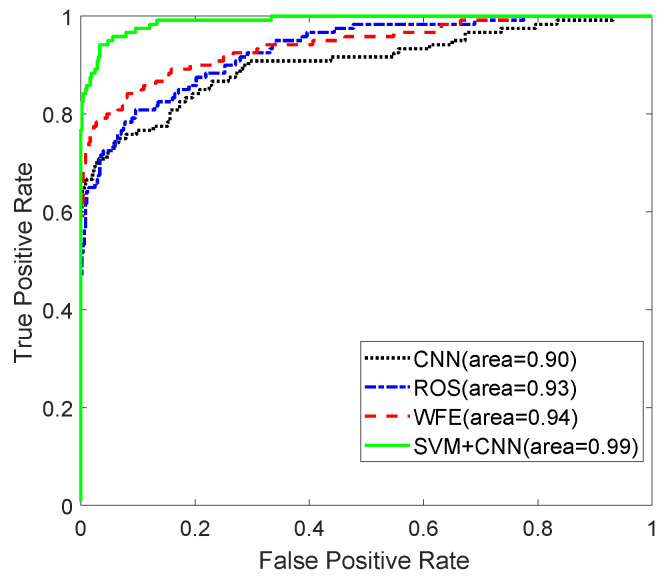
Reciever operating characteristic (ROC) curves.

In the CNN training procedure, the region-of-interest (ROI) in the Range-Doppler images had a size of 28 by 28, which corresponds to the range width of 2.8 m and the velocity width of 4.5 m/s, is clipped as input to the CNN classifier. In the modified downscaled CNN, the weight $w_{1}$ is set to 0.2, and then the $w_{2}$ is 0.8 in the loss function. In addition, the dropout with 50% is applied in the fully connected layer with the consideration of small-scale datasets. In the training process of the network, a Gaussian-distributed random variable is used to initialize network parameters. The learning rate is 0.001, the batch size is 120, and the gradient descent is adopted to update the network parameters. The classification results. It is observed that the proposed SVM-CNN method has the largest area under the curve (AUC) of ROC even up to 0.99, and the highest $F_{1}$ score of 0.90, as provided in Table 4 and shown in Figure 5. The corresponding classification accuracy is 0.96, the precision α is 0.92, and the recall β of 0.88, compared to the standard CNN, CNN based the ROS technique, and the WFE based CNN method which directly put the ROI images as inputs into the WFE based CNN without the SVM technique, as shown in Table 4. It is also found that the WFE-based CNN method has a slightly better performance than the standard CNN and the CNN based ROS technique, owing to bringing the WFE into the loss function in this class-imbalance case. It is found that the standard CNN method acquires the highest precision α=1 and the lowest recall β=0.62. It means that no vehicle sample is classified as pedestrian, while a number of pedestrian samples are wrongly classified as vehicle. The reason may be that the network weight updates being driven primarily by the majority class often tend to cause the majority of class errors to reduce rapidly at the expense of increasing minority class error in the standard CNN method. Therefore, the standard CNN method does not seem a good option in the class-imbalance classification task. The corresponding execution time is provided in Table 5. It is observed that the running time of the proposed SVM-CNN is comparable to the standard CNN and WFE methods, whereas the ROS method has the highest running time, owing to the random over-sampling technique for duplicating minority samples in the training set.

**Figure 6:**
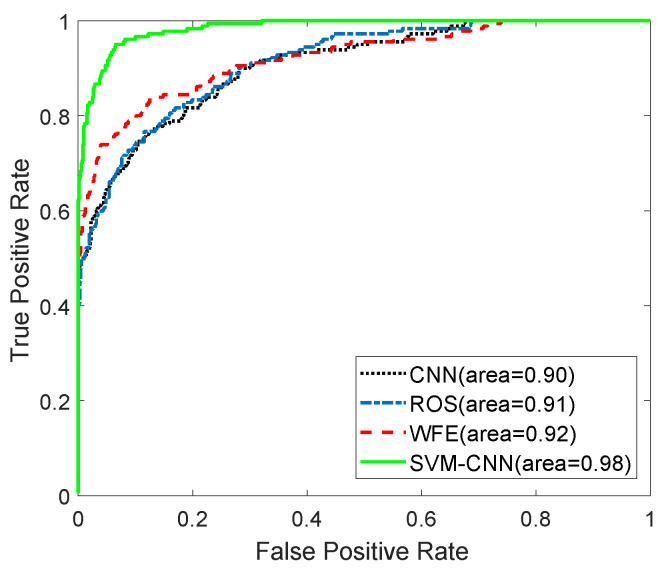
ROC curves in the mixed dataset.

To verify the effectiveness and adaptation of the proposed hybrid SVM-CNN method in the class-imbalance classification task, we take more complex scenarios in the automotive radar system into consideration. However, it is difficult for us to acquire diverse real radar data, due to the complexity and diversity of observed scenarios in practical autonomous vehicles. Fortunately, with respect to the demand of autonomous vehicles, a number of efforts have been devoted to address this issue, and many simulation models of vehicle and pedestrian in the radar system had been introduced and provided [32,46,47,48]. In order to acquire radar echo data in the diverse complex scenarios, we adopt the simulation models provided by [46,47]. In this subsection, the mixed dataset including the simulated echo data with the more complex scenarios and real experimental samples above will be used. In the simulations, the complex scenarios in the autonomous vehicles, such as, diverse observed orientations from $0^{\circ }$ to $120^{\circ }$, absolute velocities of vehicle from 4 m/s to 30 m/s and of pedestrian from 0.5 m/s to 2.0 m/s, slow down operation and acceleration operation, are respectively modeled in the radar system. 3000 samples are simulated and generated with the number of vehicles of 2500 and the number of pedestrians of 500 in the diverse and complex scenarios. Therefore, the mixed dataset with the total sample number of 6000 including 3000 experimental samples and 3000 simulated samples is used to perform the subsequently performance analysis. The simulated parameters follow these in real radar system in Table 2. It is observed that the proposed SVM-CNN method acquires the the largest AUC of 0.98 and the highest $F_{1}$ score of 0.86, compared to these of 0.92 and 0.76, 0.92 and 0.67, 0.91 and 0.62 in the WFE, ROS and standard CNN methods, respectively, even in the complex scenarios, as provided in Table 6 and shown in Figure 6. It is found that the standard CNN method has the lowest $F_{1}$ score and AUC among these methods, although it has the highest precision α=1. The value of the recall β=0.45 suggests the number of pedestrians wrongly classified as vehicles is the highest among these methods.

## 5. Conclusions

Human–vehicle classification is always an essential component to avoid accidents in autonomous driving, and has been paid more attention by related researchers. In a practical automotive context, it often occurs that the number of detected vehicles among detections is much more than the number of pedestrians, and it would lead to a typical class imbalance issue. In this paper, a hybrid support vector machine–convolutional neural network (SVM-CNN) method was proposed to perform the class-imbalance classification of vehicles and pedestrians with limited experimental radar data available in an automotive radar sensor. A two-stage scheme with the combination of feature-based SVM technique and deep learning-based CNN was employed. In the first stage, the prior thresholds of velocity and range extension were used to relearn the intercept, the modified SVM technique based on these distinct physical features was used to recognize vehicles to effectively alleviate the imbalance ratio of vehicles to pedestrian in the data level. The residual unclassified region-of-interest Range-Doppler images will be used as inputs to the CNN for the subsequent classification. In the second stage, we introduced a weighted false error function into the loss function to pay more attention to the minority, and took full advantage of the powerful ability of automatic feature learning in the CNN to improve the classification performance in the algorithm level. Experimental results verified superior performances of the proposed method over several state-of-the-art methods with limited experimental radar data available in a 77 GHz automotive radar sensor.

## Figures and Tables

**Table 1:** Feature vector.

Feature	Description
$R_{\mathrm{profile}}$	Range extension
$R_{\mathrm{var}}$	Variance estimation in range
$v_{r}$	Radial velocity
$v_{\mathrm{profile}}$	Velocity extension
$v_{\mathrm{var}}$	Variance estimation in $v_{r}$

**Table 2:** Radar parameters.

Parameters	Value
Number of sample per chirp	250
Number of chirps per frame	128
Chirp bandwidth	1500 MHz
Chirp duration	100 µs
Frequency slope	30 MHz/µs
Carrier frequency	77 GHz
ADC sampling frequency	10 MHz
Transmitter-receiver(TX/RX) channels	1/4

**Table 3:** Results of the SVM classifiers.

		The SVM Classifier	The Improved SVM Classifier
The training set	Number of samples classified as vehicles	1066	483
	Number of vehicle samples classified correctly	923	483
	Precision	0.87	1
The test set	Number of samples classified as vehicles	268	126
	Number of vehicle samples classified correctly	228	126
	Precision	0.85	1

**Table 4:** Classification result comparisons. Bold values in the table denote the highest one in each attribute.

Method	Accuracy	Precision (α)	Recall (β)	$F_{1}$ Score	AUC
CNN	0.92	**1.00**	0.62	0.75	0.90
ROS	0.90	0.72	0.78	0.75	0.93
WFE	0.94	0.92	0.76	0.83	0.94
SVM-CNN	**0.96**	0.92	**0.88**	**0.90**	**0.99**

**Table 5:** Comparisons of execution time.

Method	CNN	ROS	WFE	SVM-CNN
Running Time (min)	15.22	23.66	16.25	18.27

**Table 6:** Classification result comparisons in the mixed dataset. Bold values denote the highest one in each attribute.

Method	Accuracy	Precision (α)	Recall (β)	$F_{1}$ Score	AUC
CNN	0.89	**1.00**	0.45	0.62	0.90
ROS	0.90	0.86	0.56	0.67	0.92
WFE	0.91	0.84	0.71	0.76	0.92
SVM-CNN	**0.95**	0.95	**0.78**	**0.86**	**0.98**

## References

[1] Dollar P., Wojek C., Schiele B., Perona P. (2011). Pedestrian detection: An evaluation of the state of the art. IEEE Trans. Pattern Anal. Mach. Intell..

[2] Enzweiler M., Gavrila D.M. (2008). Monocular pedestrian detection: Survey and experiments. IEEE Trans. Pattern Anal. Mach. Intell..

[3] Cordts M., Omran M., Ramos S., Rehfeld T., Enzweiler M., Benenson R., Franke U., Roth S., Schiele B. The cityscapes dataset for semantic urban scene understanding. Proceedings of the IEEE Conference on Computer Vision and Pattern Recognition.

[4] Bilik I., Tabrikian J., Cohen A. (2006). GMM-based target classification for ground surveillance Doppler radar. IEEE Trans. Aerosp. Electron. Syst..

[5] Heuel S., Rohling H. Two-stage pedestrian classification in automotive radar sensors. Proceedings of the 12th International Radar Symposium (IRS).

[6] Schubert E., Meinl F., Kunert M., Menzel W. Clustering of highresolution automotive radar detections and subsequent feature extraction for classification of road users. Proceedings of the 16th International Radar Symposium (IRS).

[7] Lee J., Kim D., Jeong S., Ahn G.C., Kim Y. (2016). Target classification scheme using phase characteristics for automotive FMCW radar. IET Electron. Lett..

[8] Lee S., Yoon Y.J., Lee J.E., Kim S.C. (2017). Human-vehicle classification using feature-based SVM in 77-GHz automotive FMCW radar. IET Radar Sonar Navig..

[9] Tahmous D. (2015). Review of micro-Doppler signatures. IET Radar Sonar Navig..

[10] Fioranelli F., Ritchie M., Griffiths H. (2017). Feature diversity for optimized human micro-Doppler classification using multistatic radar. IEEE Trans. Aerosp. Electron. Syst..

[11] LeCun Y., Bengio Y., Hinton G. (2015). Deep learning. Nature.

[12] Jokanovic B., Amin M. (2018). Fall detection using deep learning in range-Doppler radars. IEEE Trans. Aerosp. Electron. Syst..

[13] Seyfioglu M.S., Gurbuz S.Z. (2017). Deep neural network initialization methods for micro-Doppler classification with low training sample support. IEEE Geosci. Remote Sens. Lett..

[14] Kim B.K., Kang H., Park S. (2017). Drone classification using convolutional neural networks with merged Doppler images. IEEE Geosci. Remote Sens. Lett..

[15] Hadhrami E.A., Mufti M.A., Taha B., Werghi N. Ground moving radar targets classification based on spectrogram images using convolutional neural networks. Proceedings of the 19th International Radar Symposium (IRS).

[16] Angelov A., Robertson A., Murray-Smith R., Fioranelli F. (2018). Practical classification of different moving targets using automotive radar and deep neural networks. IET Radar Sonar Navig..

[17] Kim W., Cho H., Kim J., Kim B., Lee S. (2020). YOLO-based simultaneous target detection and classification in automotive FMCW radar systems. Sensors.

[18] Trommel R.P., Harmanny R.I.A., Cifola L., Driessen J.N. Multi-target human gait classification using deep convolutional neural networks on micro-Doppler spectrograms. Proceedings of the European Radar Conference.

[19] Klarenbeek G., Harmanny R.I.A., Cifola L. Multi-target human gait classification using LSTM recurrent neural networks applied to micro-Doppler. Proceedings of the European Radar Conference (EURAD).

[20] Krawczyk B. (2016). Learning from imbalanced data: Open challenges and future directions. Prog. Artif. Intell..

[21] Hensman P., Masko D. (2015). The Impact of Imbalanced Training Data for Convolutional Neural Networks. http://urn.kb.se/resolve?urn=urn:nbn:se:kth:diva-166451.

[22] Pouyanfar S., Tao Y., Mohan A., Tian H., Kasseb A.S., Gauen K., Dailey R., Aghajanzadeh S., Lu Y.-H., Chen S.-C. Dynamic sampling in convolutional neural networks for imbalanced data classification. Proceedings of the IEEE Conference on Multimedia Information Processing and Retrieval (MIPR).

[23] Buda M., Maki A., Mazurowski M.A. (2018). A systematic study of the class imbalance problem in convolutional neural networks. Neural Netw..

[24] Lin T.Y., Goyal P., Girshick R., He K., Dollar P. Focal loss for dense object detection. Proceedings of the IEEE International Conference on Computer Vision (ICCV).

[25] Wang H., Cui Z., Chen Y., Avidan M., Abdallah A.B., Kronzer A. (2018). Predicting hospital readmission via cost-sensitive deep learning. IEEE/ACM Trans. Comput. Biol. Bioinf..

[26] Liu X., Wu J., Zhou Z. (2009). Exploratory undersampling for class-imbalance learning. IEEE Trans. Syst. Man. Cybern. Part B (Cybern.).

[27] Mease D., Wyner A.J., Buja A. (2007). Boosted classifcation trees and class probability quantile estimation. J. Mach. Learn. Res..

[28] Winkler V. Range Doppler detection for automotive FMCW radars. Proceedings of the European Radar Conference.

[29] Carrara G.W., Goodman S.R., Majewski M.R. (1995). Spotlight Synthetic Aperture Radar: Signal Processing Algorithms.

[30] Magaz B., Belouchrani A. (2011). Automatic threshold selection in OS-CFAR radar detection using information theoretic criteria. Prog. Electromagn. Res..

[31] Ester M., Kriegel H.P., Sander J., Xu X. (1996). A density-based algorithm for discovering clusters in large spatial databases with noise. KDD-96 Proceedings.

[32] Schubert E., Kunert M., Menzel W., Frischen A. A multi-reflection-point target model for classification of pedestrians by automotive radar. Proceedings of the 11th European Radar Conference (EuRAD).

[33] Platt J. (1998). Sequential Minimal Optimization: A Fast Algorithm for Training Support Vector Machines.

[34] He H., Garcia E.A. (2009). Learning from imbalanced data. IEEE Trans. Knowl. Data Eng..

[35] Kim Y., Moon T. (2016). Human detection and activity classification based on micro-Doppler signatures using deep convolutional neural networks. IEEE Geosci. Remote Sens. Lett..

[36] Jokanovic B., Amin M.G., Ahmad F. Effect of data representations on deep learning in fall detection. Proceedings of the IEEE Sensor Array and Multichannel Signal Processing Workshop (SAM).

[37] Jokanovic B., Amin M., Erol B. Multiple joint-variable domains recognition of human motion. Proceedings of the IEEE Radar Conference (RadarConf).

[38] Krizhevsky A., Sutskever I., Hinton G.E. Imagenet classification with deep convolutional neural networks. Proceedings of the Advances in Neural Information Processing Systems.

[39] Leem S.K., Khan F., Cho S.H. (2019). Detecting mid-air gestures for digit writing with radio sensors and a CNN. IEEE Trans. Instrum. Meas..

[40] Arsalan M., Santra A. (2019). Character recognition in air-writing based on network of radars for human-machine interface. IEEE Sens. J..

[41] Khan F., Leem S.K., Cho S.H. (2020). In-air continuous writing using UWB impulse radar sensors. IEEE Access.

[42] Anand R., Mehrotra K.G., Mohan C.K., Ranka S. (1993). An improved algorithm for neural network classification of imbalanced training sets. IEEE Trans. Neural Netw..

[43] Burges C.J. (1998). A tutorial on support vector machines for pattern recognition. Data Min. Knowl. Discov..

[44] He K., Sun J. Convolutional neural networks at constrained time cost. Proceedings of the IEEE Conference on Computer Vision and Pattern Recognition (CVPR).

[45] TI IRW1443 Evaluation Board. http://www.ti.com/tool/IWR1443BOOST.

[46] He F., Huang X., Liu C., Zhou Z., Fan C. Modeling and simulation study on radar Doppler signatures of pedestrian. Proceedings of the IEEE Radar Conference.

[47] Schubert E., Kunert M., Menzel W., Fortuny-Guasch J., Chareau J.M. Human RCS measurements and dummy requirements for the assessment of radar based active pedestrian safety systems. Proceedings of the 14th International Radar Symposium (IRS).

[48] Geary K., Colburn J.S., Bekaryan A., Zeng S., Litkouhi B., Murad M. Automotive radar target characterization from 22 to 29 GHz and 76 to 81 GHz. Proceedings of the IEEE Radar Conference (RadarCon13).

